# Anti-Vaccinationists and Modified Smallpox

**Published:** 1909-07-31

**Authors:** 


### Anti-Vaccinationists and Modified Smallpox.

The Secretary of the National Anti-Vaccination League has sent us a marked copy of its official organ, the *Vaccination Inquirer*, with a letter asking us to study the extracts there printed from the report of the Medical Officer of Health for Leicester. The moderation and courtesy which are displayed in this note we acknowledge with pleasure; we could wish that the same might be said of the contents of the journal which has been forwarded to us. On reading through it there appear on every page, almost in every paragraph, those peculiar misconceptions and mistakes which arise inevitably when ignorant and prejudiced people begin to talk at large about a very intricate science, such as that of medicine, of which their practical acquaintance is absolutely nothing. The *Inquirer* abounds in the familiar denunciations of vaccination and of all forms of serum or vaccine therapy as " filthy iniquity," " loathsome art," " unclean fraud," and the like; and does not scruple to insinuate and to assert broadcast that vaccination is upheld by the medical profession solely because it affords fees, and in spite of the fact that it causes great suffering and much intercurrent disease. It is this sort of thing which makes impartial people, whether doctors or not, decline to take seriously the anti-vaccinators. Do let the ground be cleared of all cant about loathsome acts and the like; let it be recognised that culture media are no more and no less filthy than consommé soup, that bacteria are no more and no less unclean than the yeast with which bread is made, and that serum is no more and no less loathsome than beefsteak. Then there will be common ground to serve as the basis for some useful discussion.

As for the marked page, dealing with Leicester and the large proportion of unvaccinated there, the Secretary of the League surely does not imagine that the extracts given from the medical officer of health's report reveal anything very novel. It is no news that the protection afforded by vaccination is temporary only, and that vaccination during infancy protects no longer than up to ten or twelve years of age. That, one would think, is chiefly an argument for re-vaccination at intervals. " Experience of smallpox in Leicester seems to show," continues Dr. Millard's report, " that it is essentially the adult population which is the principal factor in the spread of the disease "; that part of the population, in fact, which is least efficiently vaccinated. The next quotation is one dealing with the difficulty of detecting the very mild variety of smallpox which is commonly exhibited when vaccinated persons do chance to acquire the disease: the remark is made that such cases are so difficult to diagnose that sometimes they actually conduce to the spread of infection for that reason. But the League cannot really have it both ways: if their interpolated headline in thick type, " Vaccinated Persons Spread the Disease," is allowable, then they must admit that modified smallpox is modified in virtue of previous vaccination, and they must also take the word of smallpox experts that vaccination not only modifies the disease to this degree, but also that it very largely protects from and prevents it altogether. We are willing, aye anxious, to consider every particle of evidence for and against vaccination: the medical profession is not committed to any preconceived opinion on the merits of this or any other form of treatment on the ground that it is or is not " filthy," but only wants to give the best advice to those who seek health and the prevention of disease. The profession is prepared some day, when the biology of smallpox has been worked out, as it assuredly will be, to adopt a more scientific prophylaxis based upon bacteriology in place of the present system of empiricism. But until then we are not prepared to let go our hold of the best anchor we have, and especially we are not to be deterred by the grossly offensive suggestion that to earn a half-crown fee medical men are willing to connive at and actually to commit what they know to be an immoral and barbarous crime.

